# Using the Bair Hugger™ temperature monitoring system in neck and chest regions: a pilot study

**DOI:** 10.1186/s40981-019-0252-z

**Published:** 2019-05-16

**Authors:** Shunsuke Tachibana, Yutaro Chida, Michiaki Yamakage

**Affiliations:** Department of Anesthesiology, Sapporo Medical University, School of Medicine, Sapporo, South 1, West 16, Chuo-ku, Sapporo, Hokkaido 060-8543 Japan

**Keywords:** Perioperative body temperature monitoring, Bair Hugger™ temperature monitoring system, Non-cardiac surgery

## Abstract

**Purpose:**

Temperature monitoring in the perioperative periods is important in order to avoid both hyperthermia and hypothermia. In our pilot study, we evaluated the usefulness of Bair Hugger™ temperature monitoring system (BHTMS), a forehead deep temperature monitoring system, in the neck and chest under general anesthesia.

**Methods:**

After approval from the Sapporo Medical University Research Ethics Board, 30 female patients scheduled for laparoscopic surgery were enrolled in this study. Patients were divided into three groups, depending on the attachment regions of BHTMS sensor. Temperatures obtained from the three regions and each esophageal temperature (T_Eso_) were monitored and analyzed.

**Results:**

A Bland-Altman plot showed that the mean bias between temperature obtained from the neck and T_Eso_ was + 0.05 °C above T_Eso_ (2SD ± 0.35 °C), and that between temperature obtained from the chest and T_Eso_ was − 0.55 °C above T_Eso_ (2SD ± 0.55 °C).

**Conclusion:**

By using the BHTMS sensor in the neck region, it is possible to monitor core body temperature seamlessly and with high reliability. These results may suggest that the use of BHTMS has high versatility in measuring perioperative core body temperature.

**Trial registration:**

This study was approved by the Sapporo Medical University Research Ethics Board (2015: No. 262-149) and registered with UMIN Clinical Trial Registry (UMIN000016802 Registered 15 March 2015).

## Background

Temperature monitoring in the perioperative periods is important in order to avoid both hyperthermia and hypothermia. Malignant hyperthermia, the phenomenon of hyperthermia during use of volatile anesthetics and succinylcholine, requires monitoring and urgent treatment for the drastic temperature elevation [1, 2]. Perioperative hypothermia is also known to be harmful to patients because it can cause adverse events such as life-threating arrhythmia [3], higher risk of surgical site infection (SSI) [4], abnormal hemostasis [5, 6], a possibility of massive hemorrhage [7], and harmful shivering [8, 9].

During surgery, core body temperature is usually monitored in the rectum, bladder, and ear. However, temperatures obtained from these body regions sometimes poorly reflect the real values and drastic changes in core body temperature [10–12]. As another way of monitoring core temperature, we may select zero-heat-flux thermometer which can measure deep temperature approximately 1–2 cm below the skin surface [13]. In Japan, we can use the Bair Hugger™ temperature monitoring system (BHTMS; 3M, St. Paul, MN, USA), which involves attachment of a thermal sensor on the forehead throughout the perioperative period. It was proved that this monitoring device is highly reliable for core body temperature monitoring [14, 15]. However, application of the BHTMS remains problematic in certain clinical situations. For example, in craniotomy and neck or face surgeries, we cannot attach the forehead sensor on patients’ frontal head region. The forehead sensor is also difficult to apply when the frontal head region is covered by the other probes such as depth of anesthesia or tissue oxygen saturation monitors.

Hence, we attached the forehead sensor to other body regions and continuously compared with the esophageal temperature which is highly reliable for core body temperature [16, 17]. The aim of this study is to evaluate the performance of BHTMS in the neck and chest in Japanese patients who were scheduled non-cardiac surgery.

## Materials and methods

This study was approved by the Sapporo Medical University Research Ethics Board (2015, No. 262-149; Chairperson, N. Masumori), and written informed consent was obtained from each participant. The trial was registered at the UMIN Clinical Trials Registry (UMIN000016802) before patient recruitment. Eligible participants were 30 female patients between 21 and 80 years old who had scheduled laparoscopic surgery under general anesthesia except for upper gastrointestinal tract surgeries. The patients enrolled in this study (American Society of Anesthesiologist physical status 1–3) have no esophageal lesions and/or no abnormalities in the region of attachment and were not expected to be suffering from massive hemorrhage during surgery. We divided the participants into three groups—the forehead group (*N* = 10), neck group (*N* = 10), and anterior chest group (*N* = 10) due to different attachment regions of BHTMS sensor. None of the patients received premedication before entering the operation room. In the operation room, a 20G intravenous catheter was inserted into the light forearm and bicarbonate Ringer’s solution fluids warming at 37.0 °C started to infuse. General anesthesia was induced by using propofol (1.5–2.0 mg/kg) and fentanyl (1.0–2.0 μg/kg) and the tracheal intubation was performed after injecting 0.9 mg/kg rocuronium. After intubation, esophageal temperature probe (Novatemp®; NOVAMED, NY, USA) was properly inserted into the esophagus under observing by McGrath®MAC (Aircraft Medical CO., LTD, UK). We located the temperature probe tip in the lower esophagus and the distance was predicted in advance by referring to patients’ chest radiograph. And then, BHTMS sensor was placed on the light forehead in forehead group, above the left common carotid artery in the neck group, and on the 4 left sternal borders in the anterior chest group. After setting the thermometers, we performed the ultrasound-guided transversus abdominis plane block or quadratus lumborum block using 0.375% ropivacaine (dose within 3.0 mg/kg) as postoperative pain control. These processes of general anesthesia induction were performed by either two experienced anesthesiologists. Anesthesia during the operation was maintained with 1.5% sevoflurane in 3 L/min air and 1 L/min oxygen with 0.1–0.2 μg/kg/min remifentanil continuous administration. The ambient temperature was maintained approximately at 23.0 °C and the humidity at 40% during operation. Approximately 60 min before the end of the surgery, 1000 mg acetaminophen was intravenously administrated. Esophageal temperature (T_Eso_) and temperature obtained from three locations (T_Head_, T_Neck_, T_Chest_) were monitored and continuously recorded to a laptop computer at 5-min intervals until just before discontinuation of sevoflurane exposure. Anesthesiologist in attendance appropriately used a forced-air warming system (Bair Hugger™ patient warming Model 750; 3M, MN, USA) and blankets (patient warming Model 522 and Model 545; 3M, MN, USA), warmed the patients at 43 °C during general anesthesia.

One-way ANOVA was used to compare the patients’ background differences of age, height, weight, and body mass index (BMI). Kruskal-Wallis test was used to compare the differences of the length of operation time, length of anesthesia time, blood loss, and estimated water balance. *P* values < 0.05 were considered to indicate significant differences. Each of T_Head_, T_Neck_, and T_Chest_ were evaluated in comparison with T_Eso_ as a core body temperature. Pearson’s correlation and Bland-Altman plots were used to compare T_Eso_ and T_Head_, T_Eso_ and T_Neck_, and T_Eso_ and T_Chest_. Bland-Altman plots were used to evaluate the limits of agreement (LOA) between T_Eso_ and T_Head_, T_Eso_ and T_Neck_, and T_Eso_ and T_Chest_. Considered to several past articles about body temperature monitoring, the mean value of the difference (bias) < 0.4 °C and 2 standard deviations (SD) < ± 1.0 °C was defined statistically significant in this study. All statistical analyses were performed using Prism software version 6 for Windows (GraphPad Software Inc., La Jolla, CA, USA).

## Results

**Fig. 1:**
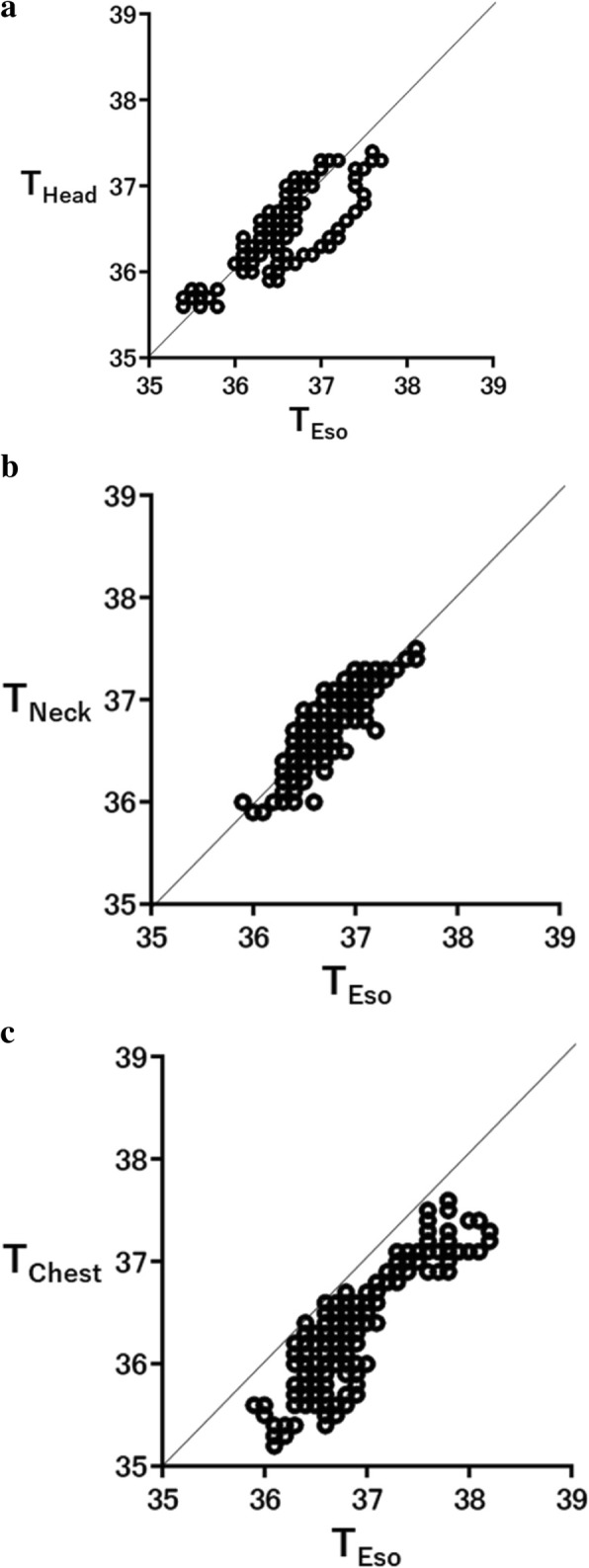
Pearson’s correlation between esophageal temperature and other temperatures. **a** Plots of temperature data obtained from the forehead (T_Head_) and esophagus (T_Eso_), **b** neck (T_Neck_) and T_Eso_, and **c** anterior chest (T_Chest_) and T_Eso_. Thin lines in the figure denote the line of identify. Pearson’s correlation coefficient indicated a strong correlation for all comparisons (*r* = 0.81 between T_Eso_ and T_Head_, *r* = 0.86 between T_Eso_ and T_Neck_, and *r* = 0.84 between T_Eso_ and T_Chest_)

We totally obtained 303, 446, and 487 data pairs for the comparisons of T_Eso_ and T_Head_, T_Eso_ and T_Neck_, and T_Eso_ and T_Chest_, respectively. No monitoring defect caused by electronic devices was observed in this study. There were no significant differences in age, height, weight, BMI, length of operation, length of anesthesia, blood loss, and estimated water balance in each group (Table 1). Figure 1 a–c show Pearson’s correlation coefficient (*r*) in each comparison. Pearson’s correlation coefficients were 0.81, 0.86, and 0.84 between T_Eso_ and T_Head_, T_Eso_ and T_Neck_, and T_Eso_ and T_Chest_, respectively, indicating a strong correlation in all comparisons (Fig. 1).

**Table 1:**
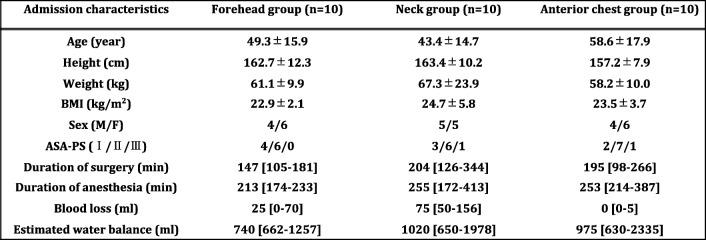
The admission characteristics of participants in each group. Data are represented as mean ± SD, medians (interquartile ranges [IQR]), or absolute number. There was no significant difference in each group statistically

*ASA-PS* American Society of Anesthesiologist physical status, *BMI* Body mass index

**Fig. 2:**
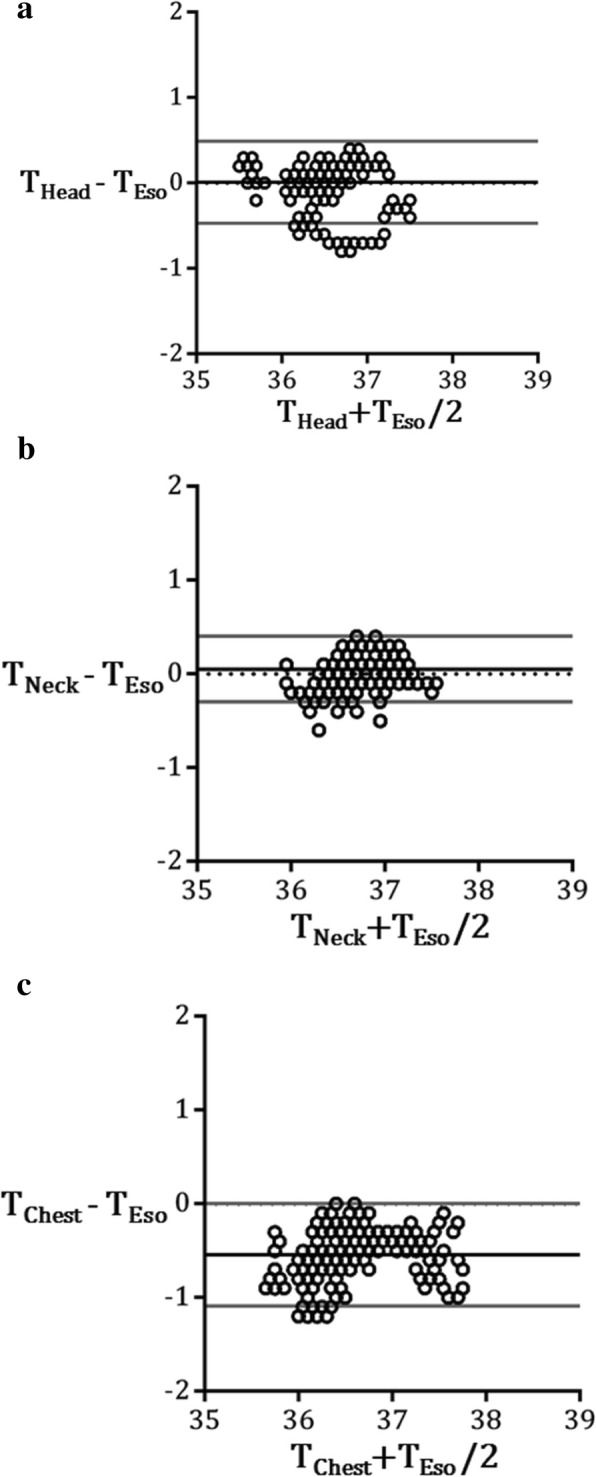
Bland-Altman plots for each comparison. **a** The mean bias of T_Head_ was + 0.01 °C above T_Eso_ (2SD ± 0.48 °C). **b** That of T_Neck_ was + 0.05 °C above T_Eso_ (2SD ± 0.35 °C). **c** That of T_Chest_ was − 0.55 °C below T_Eso_ (2SD ± 0.55 °C)

Figure 2a–c show the Bland-Altman plot between T_Eso_ and the other temperature values. The mean bias of BHTMS with normal forehead usage was + 0.01 °C above the T_Eso_ with ± 0.48 °C 2SD. The mean biases of T_Neck_ and T_Chest_ were + 0.05 °C above T_Eso_ with 2SD of ± 0.35 °C, and − 0.55 °C below T_Eso_ with 2SD of ± 0.55 °C, respectively (Fig. 2). There were no complications related to the location of esophageal probe insertion and of BHTMS sensor attachment in any of the cases.

## Discussion

Perioperative temperature monitoring is an essential factor as with other vital signs—blood pressure, heart rate, and percutaneous oxygen saturation. For accurate temperature measurements, we must select the kind of thermometers, insertion, or attachment regions [18, 19]. Perioperative hypothermia causes a higher risk of SSI [4], massive hemorrhage [7], and postoperative shivering [8, 9]. For prevention of evitable hypothermia during surgery, we must accurately monitor core body temperature and effectively perform body warming.

Usefulness of zero-heat-flux thermometer has been already reported in perioperative temperature monitoring. Fox and Solman first reported a principle of an electronic servo-controlled system to achieve almost complete thermal insulation [20]. Zero-heat-flux thermometer sensor contains two thermistors, separated by a thermal insulator and covered by an electric heater. BHTMS, one of the zero-heat-flux thermometers, also has some advantages to use in its accuracy, ease of use, and disposable sensor compared with former types. Eshraghi showed that the overall difference between the temperature obtained from BHTMS and that of the pulmonary artery was − 0.23 °C (95% LOA of ± 0.82 °C) [14]. However, this thermometer is unsuitable for use in certain clinical situations. In patients undergoing craniotomy, it is impossible to attach the BHTMS sensor to the patients’ forehead. During surgery of the neck and face, as well, surgeons do not permit the BHTMS sensor to be attached due to the proximity of the forehead to the surgical site. Use of a BIS monitor sensor and/or INVOS™ sensor on the patients’ forehead is common during cardiovascular surgery, which interferes with the attachment of the BHTMS sensor on the forehead. Since temperature monitoring is more significant in the surgeries described above, an alternative method to use the BHTMS for the measurement of core body temperature is required. Judging from the principle of the zero-heat-flux thermometer, we hypothesized that core body temperature would be measurable by attaching the BHTMS sensor to other areas with good vascularity. In this pilot study, we could prove a high correlation and accurate performance in the neck group. Eshraghi also presented that bias and precision values for neck site were similar to the forehead values [14]. It was almost comparable performance between standard forehead attachment and neck attachment. Furthermore, the systematic error was absent in the neck group. There are several reasons why core temperature monitoring in the neck is appropriate. The neck is anatomically close to the heart and is a route to the cerebral blood flow; therefore, the blood temperature hardly decreases. Besides, blood vessels in the neck run 1–2 cm below the skin, which has little influence by fatty tissues, making it easier to measure core temperature accurately.

Meanwhile, the mean bias was − 0.55 °C in the chest group, which had a systematic error. LOA in this systematic error ranged from − 1.04 °C (lower coefficient limit) to − 0.06 °C (upper coefficient limit), it is necessary to consider how to interpret numerical values under real clinical use. One reason for systematic error with anterior chest application might be influenced by the effect of the rib bones and pericardial fatty tissue, and movements of the thoracic cage and aerated lung during breathing.

Our study has certain limitations. First, we monitored and analyzed core body temperature within a limited range, almost between 35.5 and 38.0 °C, in laparoscopic surgery cases. Thus, we cannot judge whether our monitoring method is proper in hyperthermia cases over 38.0 °C and hypothermia cases below 35.5 °C. Second, we collected 10 cases and data from each group, but the sample size was too small. Third, we did not measure peripheral temperature in this study, to serve as a comparison. We need further verifications to resolve these limitations; however, the knowledge obtained from this study would be one option in monitoring core body temperature.

## Conclusions

To conclude, it is possible to monitor core body temperature seamlessly and with high reliability by using the BHTMS in the neck region. Usefulness of BHTMS in the neck is equivalent to that in the original frontal head region. The value measured in the chest has a systematic error, and it is necessary to judge by considering the LOA. In addition, these results from this pilot study suggest that using BHTMS may be more versatile in measuring perioperative core body temperature.

## Abbreviations

BHTMS: Bair Hugger™ temperature monitoring system
BMI: Body mass index
LOA: Limits of agreement
T_Chest_: Temperature obtained from the anterior chest
T_Eso_: Esophageal temperature
T_Head_: Temperature obtained from the forehead
T_Neck_: Temperature obtained from the neck
